# Characterization of Second-Order Reflection Bands from a Cholesteric Liquid Crystal Cell Based on a Wavelength-Swept Laser

**DOI:** 10.3390/s20164643

**Published:** 2020-08-18

**Authors:** Soyeon Ahn, Myeong Ock Ko, Jong-Hyun Kim, Zhongping Chen, Min Yong Jeon

**Affiliations:** 1 Department of Physics, Chungnam National University, 99 Daehak-ro Yuseong-gu, Daejeon 34134, Korea; ahnsoyen5@naver.com (S.A.); jxk97@cnu.ac.kr (J.-H.K.); 2 Core Technology R&D Team, Samsung Electronics, Hwaseong-si, Gyeonggi-do 18448, Korea; myeongock08@naver.com; 3 Instituted of Quantum Systems (IQS), Chungnam National University, 99 Daehak-ro Yuseong-gu, Daejeon 34134, Korea; 4 Beckman Laser Institute, UC Irvine, Irvine, CA 92612, USA; z2chen@uci.edu

**Keywords:** fiber laser, wavelength-swept laser, cholesteric liquid crystal, bandpass filter

## Abstract

We report the results of an experimental study of the characterization of second-order reflection bands from a cholesteric liquid crystal (CLC) cell that depends on the applied electric field, using a wide bandwidth wavelength-swept laser. The second-order reflection bands around 1300 nm and 1500 nm were observed using an optical spectrum analyzer when an electric field was applied to a horizontally oriented electrode cell with a pitch of 1.77 μm. A second-order reflection spectrum began to appear when the intensity of the electric field was 1.03 V_rms_/μm with the angle of incidence to the CLC cell fixed at 36°. The reflectance increased as the intensity of the electric field increased at an angle of incidence of 20°, whereas at an incident angle of 36°, when an electric field of a predetermined value or more was applied to the CLC cell, it was confirmed that deformation was completely formed in the liquid crystal and the reflectance was saturated to a constant level. As the intensity of the electric field increased further, the reflection band shifted to a longer wavelength and discontinuous wavelength shift due to the pitch jump was observed rather than a continuous wavelength increase. In addition, the reflection band changed when the angle of incidence on the CLC cell was changed. As the angle of incidence gradually increased, the center wavelength of the reflection band moved towards shorter wavelengths. In the future, we intend to develop a device for optical wavelength filters based on side-polished optical fibers. This is expected to have a potential application as a wavelength notch filter or a bandpass filter.

## 1. Introduction

Cholesteric liquid crystals (CLCs) or chiral nematic liquid crystals (NLCs) are liquid crystals (LCs) with chiral dopants that induce a periodic helical structure. They exhibit a helical structure in which the directors of the LCs are twisted and arranged in layers along the spiral axis. The details of the optical properties of cholesteric liquid crystal according to the applied electric field have been discussed for a long time [1,2,3]. The distance by which the director of the LC is rotated 360° in the axial direction of the helical is called a pitch. When the polarization direction of the incident light has the same handedness of the helical structure and the periodicity of the pitch satisfies the Bragg condition, the incident lights have selective reflection characteristics. For normal incidence, the reflection wavelength $\lambda_{o}$ is given by [4,5,6]:

(1) $$\lambda =\lambda_{o}=\bar{n}P$$

where $\bar{n}$ is the average of the ordinary ($n_{o}$) and extraordinary ($n_{e}$) refractive indices of the NLC and *P* is the pitch of the CLC. When a broadband light source is incident to the CLC cell, the reflection band Δλ can be formed when the Bragg condition is satisfied as follows:

(2) $$\Delta \lambda =\Delta nP=\left(n_{e}-n_{o}\right)P$$

where $\Delta n=\left(n_{e}-n_{o}\right)$ is the birefringence. The periodic helical structure of the CLC cell can be changed by various external stimuli such as heat, electric fields or magnetic fields [7,8,9]. Due to these characteristics, CLCs have been studied regarding the fabrication of optical devices for various applications such as liquid crystal displays (LCDs), dye lasers, notch filters, optical sensors and mirrors [10,11,12,13,14,15,16,17,18,19,20,21,22,23]. However, when the CLC cell is inclined with respect to the incident beam the reflection band shifts to a shorter wavelength due to the Bragg condition and a higher order reflection band appears [12,24]. For an inclined CLC cell, the center wavelength due to the Bragg reflection is given by:

(3) $$m\lambda =\bar{n}P\cos\Theta$$

(4) $$\Theta =\sin^{-1}\left(\frac{1}{\bar{n}}\sin\theta \right)$$

where θ is the angle of the incidence of the beam in the direction normal to the CLC cell and Θ is the angle between the direction of propagation and helical axis [25]. Higher order reflection bands have a much narrower bandwidth than the first-order reflection band so this phenomenon is useful for fabricating optical bandpass filters [12]. When an electric field is applied perpendicular to the helical axis of an LC having positive anisotropy, the deformation occurs because the direction of the LC is aligned in the field direction and thus the pitch increases as the deformation increases. It is known that the higher order reflection band originates from the non-sinusoidal distribution of a refractive index caused by the in-plane-field-induced distortion of the CLC helices [12]. In particular, in the case of CLCs, an electric field or magnetic field applied perpendicular to the helical axis produces a non-uniform twist of the helical structure and increases birefringence of the CLC. Chou et al. calculated the wave equation to determine the transmission coefficient of the CLC and when an electric field was applied, a second reflection appeared, which explains why the birefringence increased [26]. In addition, Dumitrascu et al. reported that when the electric field was applied perpendicular to the helical axis, the ordinary refractive index $n_{o}$ was hardly affected by the electric field but the extraordinary refractive index $n_{e}$ increased as the intensity of the electric field increased. In addition, saturation occurred in reflectance above some electric fields [27]. Therefore, it can be explained that the increase in reflectance is due to the increase in the birefringence of the LC when an electric field is applied [12,26,27].

A broadband light source is required to measure the wide reflection band in the infrared region. Wavelength swept lasers (WSLs) in the 1300 nm band or 1500 nm band have a wide scanning bandwidth of ~100 nm or wider, which is suitable for measuring these characteristics [28,29,30]. Moreover, by combining the two wavelength bands, it is possible to observe the reflection band in a much wider band region. Furthermore, it is a light source that is easy to observe regarding dynamic variations in reflection bands, with respect to changes in the electric field intensity [30].

In this paper, we successfully observed second-order reflection bands from a CLC cell that varied dependent on the applied electric field using a wide bandwidth WSL. As the intensity of the electric field was increased further, the reflectance of the CLC cell also increased. In addition, changes in the transmission spectrum were observed in response to changing the angle of the beam incident on the CLC cell under the application of a constant electric field.

## 2. Fabrication of the CLC Cell

**Figure 1:**
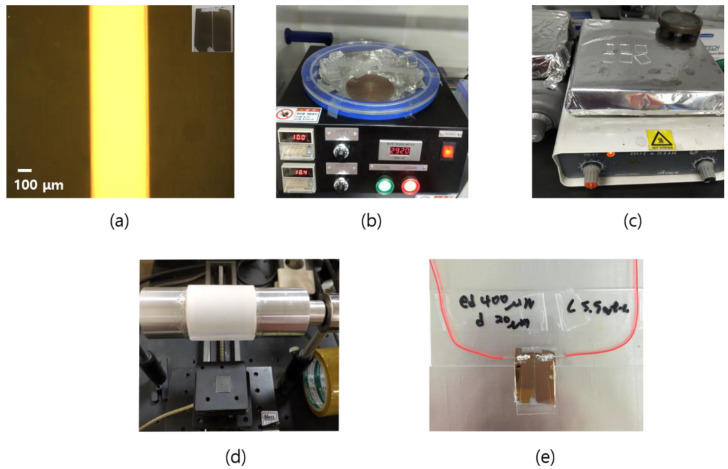
Fabrication process of the CLC cell; (**a**) electrode substrates, (**b**) spin coating, (**c**) baking, (**d**) rubbing and (**e**) the fabricated CLC cell.

In the experiments, an NLC E7 (Qingdao QY liquid crystal) and a chiral dopant R811 were mixed to produce a right-handed CLC. The ordinary and extraordinary refractive indices of the CLC were 1.5014 and 1.6885, respectively. Figure 1 shows the fabrication process of the CLC cell. First of all, the cell was prepared by cutting the slide glass to a size of ~13 mm × 18 mm using a diamond knife. The cells were then washed for 15 min in an ultrasonic cleaner in the order of de-ionized water, acetone and ethanol. Figure 1a shows an electrode substrate coated with 8 nm and 20 nm of Ti and Au, respectively, by masking the optical fiber with Kapton tape on the cleaned glass and then using an electron beam (e-beam) vacuum evaporator. A thin coating of Ti before Au on the slide glass substrate helped the Au adhere well to it. The gap between the two in-plane electrodes was ~400 μm. The electrode substrate was then washed and dried again. The polyimide solution of AL-3046 was spread onto the substrates using a pipette; it was then spin-coated at approximately 3000 rpm for 30 s (Figure 1b) and then baked for 1 h at 180 °C on a hot plate (Figure 1c). The substrate was then rubbed ~20 times in a specific direction using a rubbing machine (Figure 1d). The rubbing machine used in this experiment was homemade and the substrate was rubbed with a velvet rubbing fabric. When rubbing the electrode substrate, it was necessary to rub in a direction parallel to the electrode. This process allowed the LC molecules to align in the rubbing direction. The rubbing direction of the two substrates was then anti-parallel and the gap between the two substrates was created using a 20 μm film spacer and epoxy. In the case of an electrode cell, one substrate became an electrode substrate and the other became a general glass substrate. Figure 1e shows an indium solder wire attached to an electrode cell. This ensured that the electric field was applied perpendicular to the helical axis. In this case, the LC was injected after indium soldering because indium soldering can affect the LC by dissolving the indium at a high temperature. In the case of E7, it is a liquid crystal phase at room temperature and becomes isotropic phase when it is about 60 °C. Therefore, the CLC cell was placed on a hot plate and mixed with a chiral dopant in an isotropic state. After that, the CLC was mixed well using a vortex mixer or stirrer and then injected between two substrates using a pipette.

**Figure 2:**
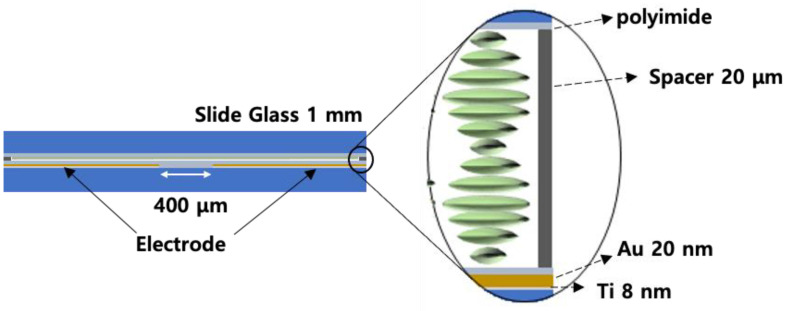
Structure of the CLC cell.

Figure 2 shows the fabricated CLC cell structure. It consisted of glass substrates, polyimide layers, electrode layers and a CLC layer. Two flat electrodes provided the in-plane electric field. The gap between the two electrodes was ~400 μm. The thickness of Au and Ti as the electrode layer together was ~28 nm and the CLC cell thickness was ~20 μm. A homogeneously aligned CLC cell was driven by an electric field perpendicular to the helical axis. Since the distance between the in-plane electrodes was sufficiently wide compared with the cell gap, it can be considered that the helical structure cell was subjected to a uniform perpendicular electric field. Since the pitch of the CLC cell changed according to the intensity of the in-plane electric field, the reflection band of the CLC cell also changed by the applied electric field [30].

**Figure 3:**
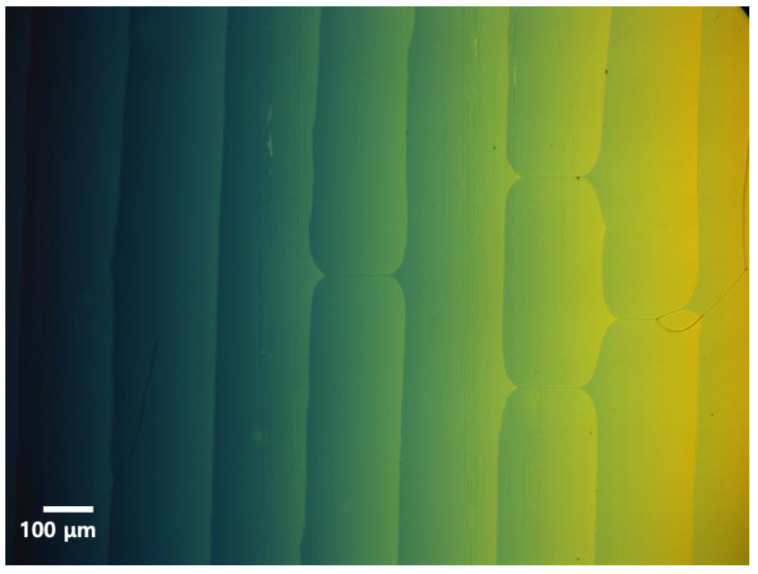
Photograph of the CLC texture.

The chiral dopant concentration of the CLC cell was 5.5 wt%. In order to measure the pitch of the CLC cell, two methods were used: one method was to measure using the Cano wedge cell having a constant slope and the other method was to measure using the transmittance spectrum from the CLC cell. The Cano wedge cell had dislocation defect walls at half-pitch because the molecules discontinuously arranged when the thickness changed by half-pitch. The pitch can be measured by the slope of the wedge cell and the distance between the dislocation lines [31]. The measurement was performed four times by the wedge cell method and the measured pitch value was taken as the average value of four measurements. Figure 3 shows the photograph of the texture for the Cano wedge cell when a CLC with a concentration of 5.5 wt% was injected into a wedge cell. The color change in the wedge cell was due to the inclination of the cell, which is a phenomenon that appears due to the difference in the cell gap. The dislocation lines were formed on the texture of the CLC Cano wedge cell.

**Figure 4:**
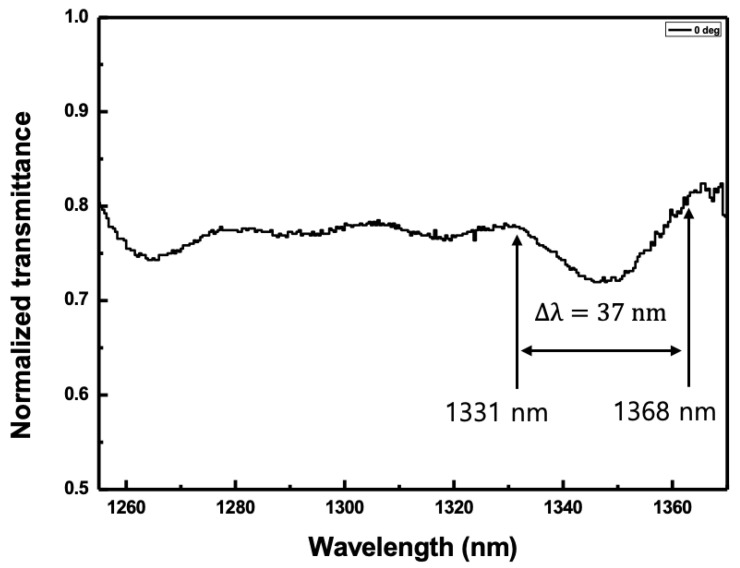
Second-order reflection spectrum when the light was incident to the normal direction of the CLC cell when the electric field applied to the CLC cell was fixed to 0.49 V_rms_/μm.

The next method was to estimate the pitch of the CLC cell using the transmittance spectrum. The WSL was positioned incident to the normal direction on the cell. The electric field applied to the CLC cell was fixed to 0.49 V_rms_/μm. The second-order reflection spectrum was achieved from 1331 nm to 1368 nm using the transmittance spectrum as shown in Figure 4. The edge-to-edge bandwidth of the reflection band was ~37 nm. The pitch was calculated to be 1.77 μm using Equation (3).

## 3. Experiments

**Figure 5:**
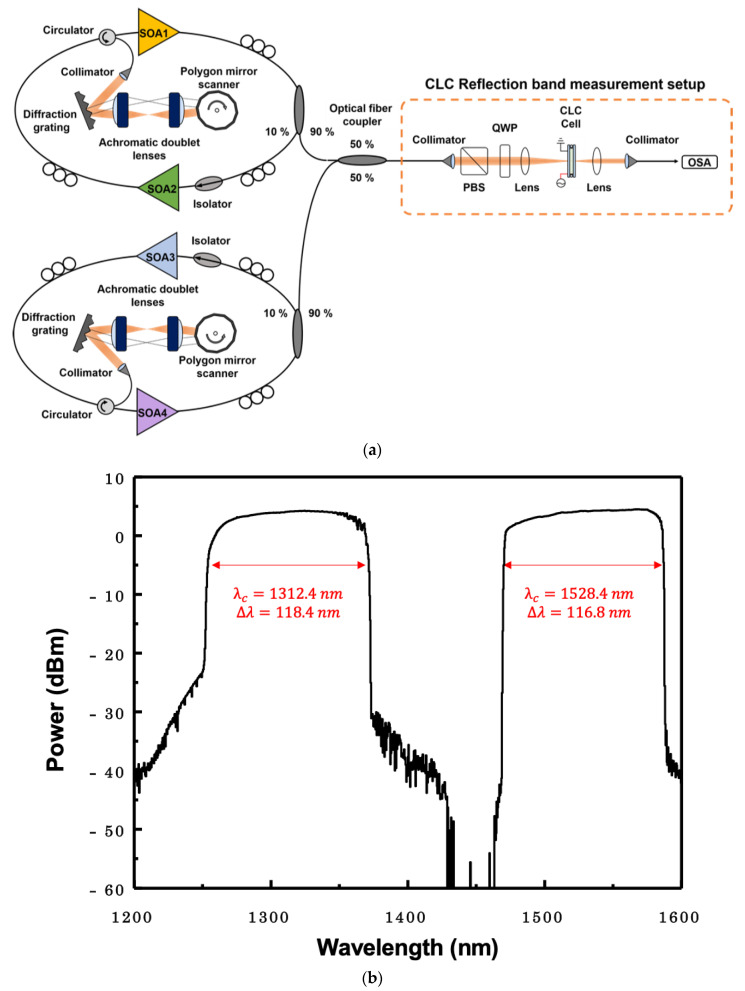
(**a**) Experimental setup for measuring the CLC reflection band and (**b**) optical spectrum output from the WSL.

The measurements of the optical characteristics of the CLC cell were carried out using a broadband WSL. With WSL light sources, wavelength characteristics can be observed over a wider range than using a semiconductor optical amplifier (SOA). Figure 5a shows the experimental setup used to measure the second-order reflection spectra of the CLC cell using two WSLs. Two WSLs were combined to form a broadband WSL from 1300 nm to 1500 nm bands. The first WSL, consisting of SOA1 and SOA2, operated in the 1300 nm band. The second WSL, consisting of SOA3 and SOA4, operated in the 1500 nm band. Each WSL consisted of two SOAs, an optical isolator, three polarization controllers, an optical output coupler, an optical circulator, a diffraction grating with 600 grooves/mm, two achromatic doublet lenses and a polygonal wavelength scanning filter [30]. The 10-dB scanning bandwidths of the WSLs around 1300 nm and 1500 nm were ~118.4 nm and ~116.8 nm, respectively, as shown in Figure 5b. The scanning range around 1300 nm and 1500 nm were from 1253.2 nm to 1371.6 nm and 1470 nm to 1586.8 nm, respectively. The scanning rate and the average output power of the WSL were 3.6 kHz and ~13 dBm, respectively. The dotted box in Figure 5a shows the measurement setup used to measure the transmittance band spectra of the CLC cell.

The output from the WSL was incident to the measurement setup. The beam was set to be right-handed circularly polarized through a polarization beam splitter (PBS) and a quarter wave plate (QWP). It was then positioned to be incident vertically on the CLC cell. The CLC cell was positioned in the depth of the focus (DOF) of the beam. The size of the beam was ~85 μm within the area of the range between the electrodes. Since the distance between the electrodes was far enough for the gap size of the CLC cell, it can be assumed that when an in-plane electric field is applied, a uniform electric field is applied to the central area of the CLC cell. The beam transmitted through the CLC cell was measured using an optical spectrum analyzer (OSA). A 5 kHz sinusoidal wave with alternating current (AC) voltage was applied to the CLC cell parallel to the surface using a function generator (Agilent) and an amplifier (Trek). The transmitted beam was measured according to the applied electric field or the angle of incidence of the CLC cell using the OSA.

**Figure 6:**
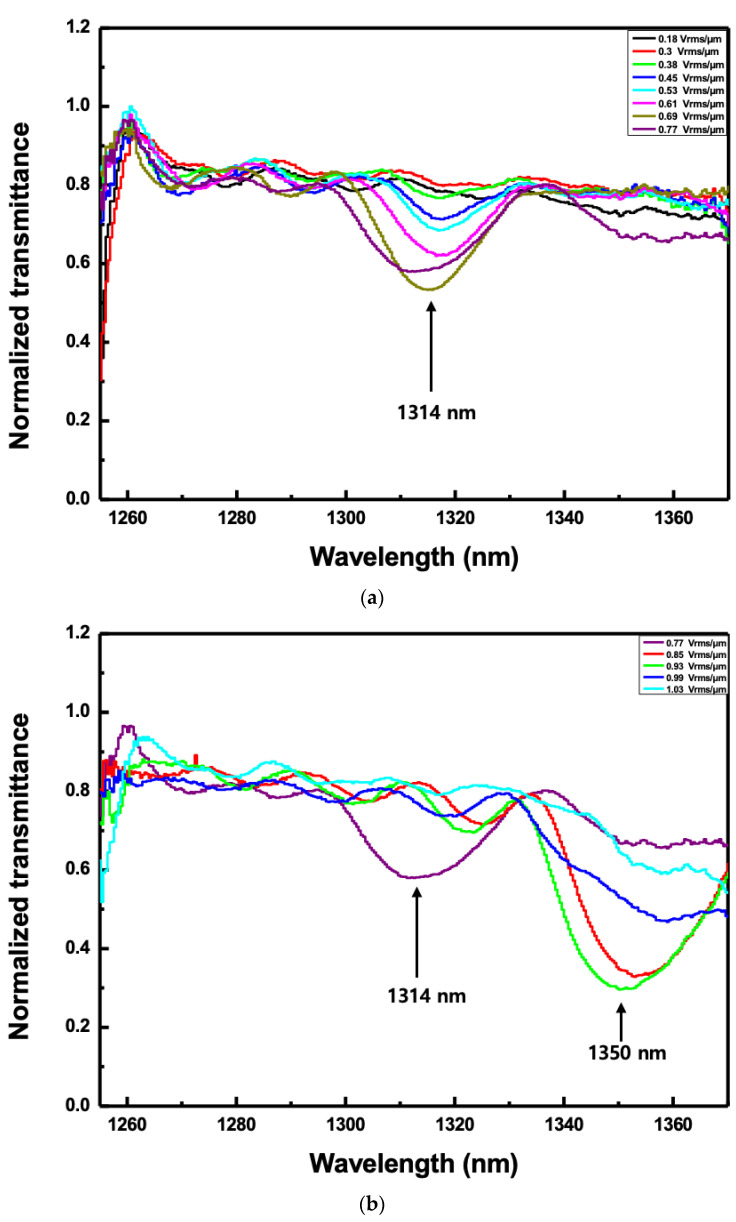
The normalized transmitted spectra according to the applied electric field when the angle of incidence to the CLC cell was fixed to 20°. (**a**) The electric field ranged from 0.18 V_rms_/μm to 0.77 V_rms_/μm and (**b**) the electric field ranged from 0.77 V_rms_/μm to 1.03 V_rms_/μm.

The voltage from 50 mV_rms_ to 270 mV_rms_ was applied to the CLC cell through the amplifier; the voltage was increased in 10 mV_rms_ steps and the corresponding electric field ranged from 0.18 V_rms_/μm to 1.03 V_rms_/μm. Figure 6 shows the normalized transmitted spectra according to the applied electric field when the angle of incidence to the CLC cell was fixed to $20^{o}$. The normalized transmitted spectra can be achieved with the following steps. First of all, in the measurement setup, the transmission spectrum was measured without the CLC cell. It can be used as a reference spectrum. After fixing the cell on the rotation stage, it was placed in the DOF region and the spectrum according to the electric field intensity or the angle of incidence was measured. The normalized transmission spectrum was obtained by differentiating this value from the reference spectrum. At the electric field of 0.36 V_rms_/μm, the second-order reflection band of the CLC cell began to appear around 1314 nm of the reflection band as shown in Figure 6a. When the electric field increased up to 0.77 V_rms_/μm, there was no change in pitch, meaning that the reflection band was almost maintained. The relative reflectance in the case of Figure 6a was measured to be ~0.3 or less. However, when it increased above 0.85 V_rms_/μm, a pitch jump occurred and the center wavelength of the reflection band moved to near 1350 nm as shown in Figure 6b. The second reflection band was maintained until the electric field was 0.99 V_rms_/μm. It was shown that the relative reflectance increased as the voltage increased. The relative reflectance in the case of Figure 6b was measured to be ~0.45 or less. When the angle of incidence was $20^{o}$, the deformation was formed as the electric field was increased so the relative reflectance tended to increase. The increase in reflectance was caused by the increase in the birefringence of the CLC when an electric field was applied [26,27].

**Figure 7:**
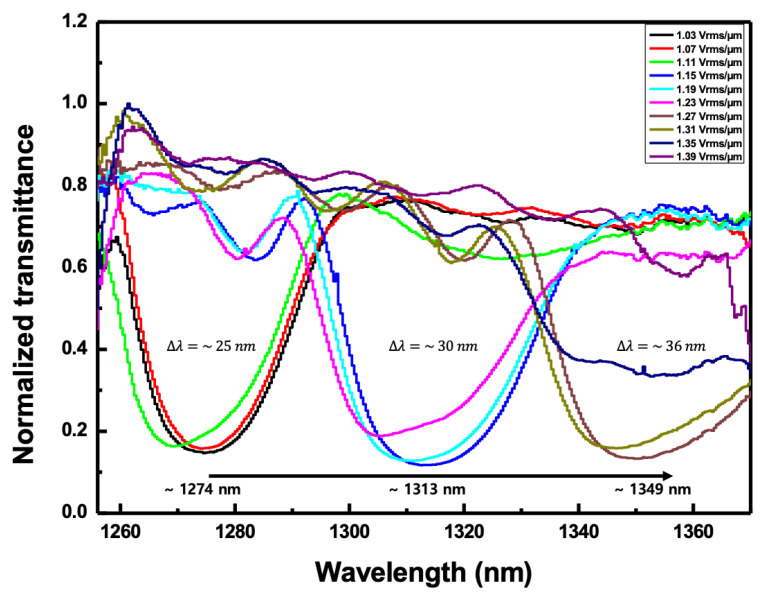
The normalized transmitted spectra according to the applied electric field when the angle of incidence to the CLC cell was fixed to 36°.

Next, the angle of incidence was changed to $36^{o}$ to observe the second-order reflection characteristics according to the applied electric field. The voltage from 270 mV_rms_ to 360 mV_rms_ was applied to the CLC cell; the voltage was increased in 10 mV_rms_ steps and the corresponding electric field ranged from 1.03 V_rms_/μm to 1.39 V_rms_/μm. Figure 7 shows the normalized transmitted spectra according to the applied electric field when the angle of incidence to the CLC cell was fixed to $36^{o}$. At the electric field of 1.03 V_rms_/μm, the second-order reflection band of the CLC cell began to appear around 1274 nm of the reflection band. When the electric field increased from 1.07 to 1.11 V_rms_/μm, there was no change in pitch. In these cases, most reflection bands had a width of ~25 nm. However, when it increased above 1.15 V_rms_/μm, a pitch jump occurred and the center wavelength of the reflection band moved to near 1313 nm. The second reflection band was maintained until the electric field was 1.23 V_rms_/μm. In these cases, most reflection bands have a width of ~30 nm. They were observed slightly wider than in the previous cases. As the intensity of the electric field increased to 1.27 V_rms_/μm, the reflection band shifted to a longer wavelength of 1349 nm. In addition, it was found that the band width was further increased to 36 nm or more. In an electric field higher than 1.39 V_rms_/μm, a pitch jump occurred and it could not be measured any more beyond the scanning wavelength range of the WSL. This discontinuous change in the reflection band indicated that the pitch of the CLC cell discontinuously varied as the intensity of the electric field increased. The discretization of the pitch according to the intensity of the electric field in the LC was strongly anchored at the surface boundary so the pitch increased discontinuously when the electric field increased to a certain value or more [12,30,32].

**Figure 8:**
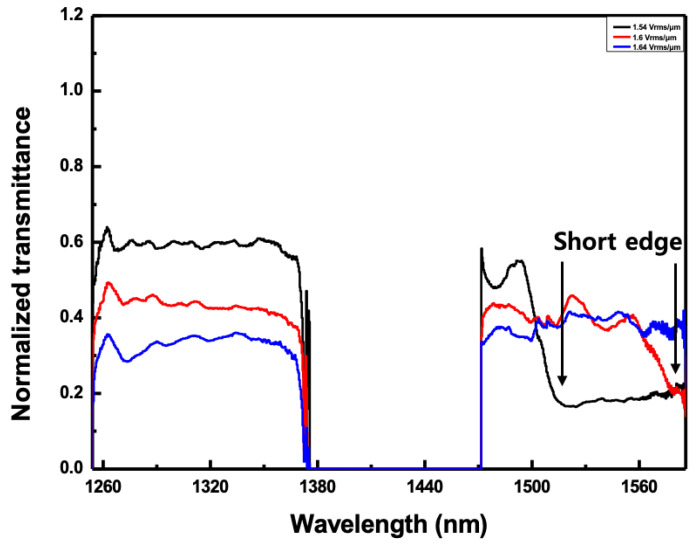
The normalized transmitted spectra according to the applied electric field more than 1.54 V_rms_/μm when the angle of incidence to the CLC cell was fixed to 36°.

In order to observe the second-order reflectance over a 1500 nm band, a higher electric field was applied to the CLC cell. Figure 8 shows the reflected spectra according to the applied electric field of more than 1.54 V_rms_/μm when the angle of incidence to the CLC cell was fixed to $36^{o}$. The full reflected band structure could not be observed because the width of the reflection bands was outside the measurable wavelength range. However, as shown in Figure 8, the short edge of the reflection band was measured when the applied electric field was more than 1.54 V_rms_/μm. When the applied electric field was 1.54 V_rms_/μm, the short edge of the reflection band appeared near 1515 nm. On the other hand, when the applied electric field was 1.6 V_rms_/μm, the short edge of the reflection band appeared near 1565 nm. However, when the applied electric field was 1.64 V_rms_/μm, the short edge of the reflection band could not be observed because it was outside the range of WSL.

**Figure 9:**
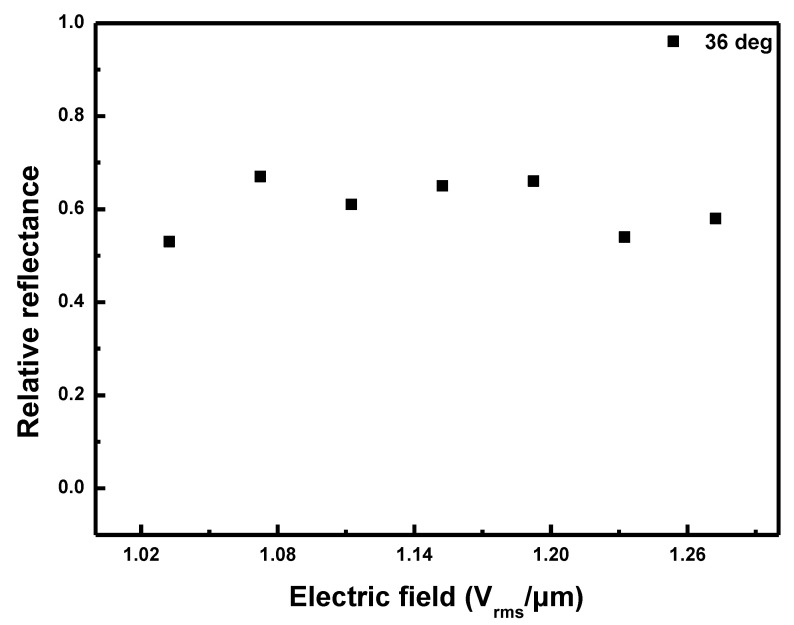
Relative reflectance of the CLC cell according to the applied electric field when the angle of incidence to the CLC cell was fixed to 36°.

Figure 9 shows the plots of the relative reflectance versus the electric field applied to the CLC cell for the reflection bands in Figure 7 when the angle of incidence to the CLC cell was fixed to $36^{o}$. The relative reflectance was obtained at values of ~0.6 as shown in Figure 8. When the angle of incidence was $20^{o}$, the relative reflectance tended to increase but at $36^{o}$, the relative reflectance was somewhat constant. This is the reason that when the intensity of the applied electric field increased above a certain value, the deformation was completely formed and thus the reflectance was saturated [27].

**Figure 10:**
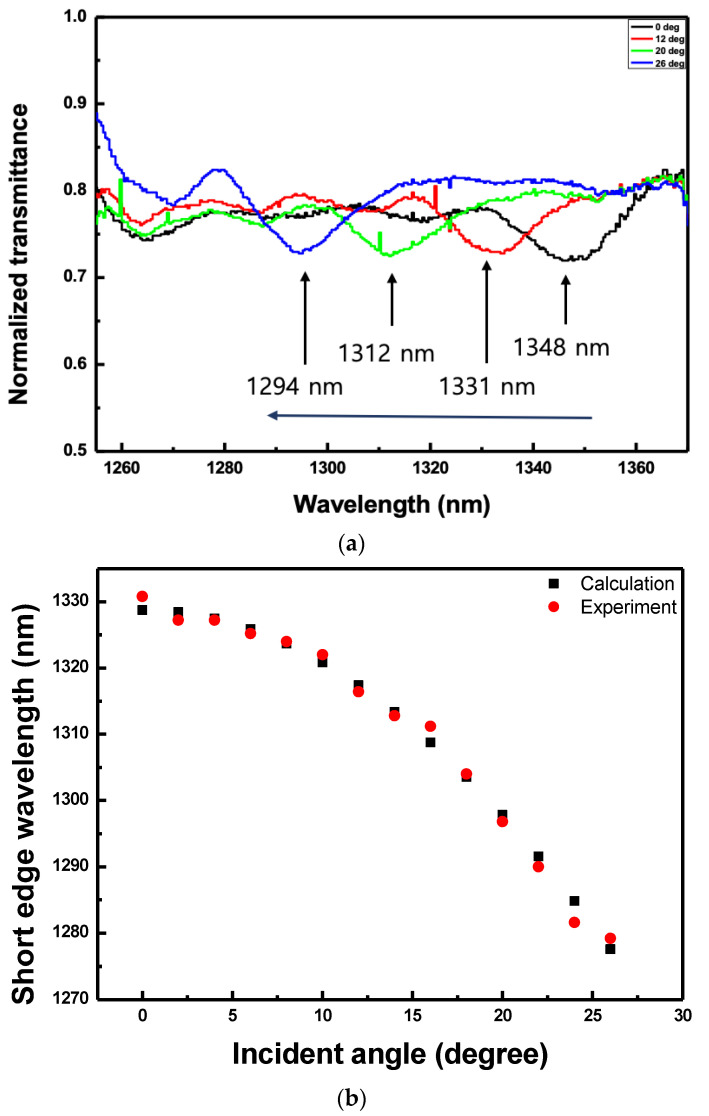
(**a**) Normalized transmitted spectra and (**b**) variation of the short edge wavelength according to the angles of incidence of the beam on the CLC cell when the electric field applied to the CLC cell was fixed to 0.49 V_rms_/μm.

Figure 10a shows the normalized transmitted spectra according to the angle of incidence to the CLC cell. The electric field applied to the CLC cell was fixed at 0.49 V_rms_/μm. The transmitted spectra were measured while inclining the CLC cell at an interval of $2^{o}$ from $0^{o}$ to $26^{o}$ with respect to the incident light. The center wavelength of the reflection band when the beam was incident vertically on the CLC cell was 1348 nm. As the angle of incidence was gradually increased, the center wavelength of the reflection band moved towards shorter wavelengths. When the angle of incidence was $12^{o}$, the center wavelength of the reflection band was 1331 nm. When it was more inclined at $20^{o}$, the reflection band moved to 1312 nm and when the angle of incidence was $26^{o}$, it moved to 1294 nm. These phenomena happened because the pitch of the CLC was different depending on the angle of incidence of the beam with respect to the CLC cell. Figure 10b shows the variation of the short edge wavelength according to the angle of incidence of the beam on the CLC cell. As the angle of inclination of the CLC cell increased, the short edge wavelength moved towards shorter wavelengths, as shown in Figure 10. The experimental data almost coincided to the value calculated using Equation (3).

## 4. Conclusions

We have successfully investigated second-order reflection bands from a cholesteric liquid crystal (CLC) cell varied dependent on the applied electric field and on the angle of incidence of the beam on the CLC cell. In order to observe the reflection spectrum, two wide-band wavelength-swept lasers (1300 nm and 1500 nm band) were used as an optical source. Second-order reflection spectra were observed using an optical spectrum analyzer after applying an electric field to a horizontally oriented electrode cell with a pitch of 1.77 μm. Second-order reflection spectra began to appear when the intensity of the electric field was 1.03 V_rms_/μm with the angle of incidence to the CLC cell fixed at $36^{o}$. As the intensity of the electric field was increased further, the reflection band shifted discontinuously towards longer wavelengths. Most of the reflection bands were about 25 nm to 36 nm, which confirmed the possibility as a wavelength bandpass filter and confirmed the possibility as an electric field sensor by using a wavelength change according to the intensity of the electric field. In addition, the reflection band changed when the angle of incidence on the CLC cell was changed under a fixed electric field. As the angle of incidence was gradually increased, the center wavelength of the reflection band moved towards a shorter wavelength. In the future, we intend to develop a device for optical wavelength filters based on side polished optical fibers. This is expected to have a potential application as a wavelength notch filter or a bandpass filter.

## References

[1] De Gennes P.-G., Prost J. (1993). The Physics of Liquid Crystals.

[2] Dunmur D., Fukuda A., Luckhurst G.R. (2001). Physical Properties of Liquid Crystals. Nematics (EMIS Datareviews Series No. 25).

[3] Goodby J.W. (1991). Chirality in liquid crystals. J. Mater. Chem..

[4] Xianyu H., Faris S., Crawford G.P. (2004). In-plane switching of cholesteric liquid crystals for visible and near-infrared applications. Appl. Opt..

[5] Mitov M., Dessaud N. (2006). Going beyond the reflectance limit of cholesteric liquid crystals. Nat. Mater..

[6] Zografopoulos D.C., Kriezis E.E., Mitov M., Binet C. (2006). Theoretical and experimental optical studies of cholesteric liquid crystal films with thermally induced pitch gradients. Phys. Rev. E.

[7] Yang D.K., West J.L., Chien L.C., Doane J.W. (1994). Control of reflectivity and bistability in display using cholesteric liquid crystals. J. Appl. Phys..

[8] Coles H., Demus D., Goodby J., Gray G.W., Spiess H.W., Vill V. (2008). Chiral Nematics: Physical properties and applications. Handbook of Liquid Crystals Set.

[9] Hrozhyk U.A., Serak S.V., Tabiryan N.V., White T.J., Bunning T.J. (2010). Optically switchable, rapidly relaxing cholesteric liquid crystal reflectors. Opt. Express.

[10] Zhu X., Ge Z., Wu T.X., Wu S.-T. (2005). Transflective liquid crystal displays. J. Disp. Technol..

[11] Schmidtke J., Jünnemann G., Keuker-Baumann S., Kitzerow H.S. (2012). Electrical fine tuning of liquid crystal lasers. Appl. Phys. Lett..

[12] Rumi M., White T.J., Bunning T.J. (2014). Reflection spectra of distorted cholesteric liquid crystal structures in cells with interdigitated electrodes. Opt. Express.

[13] Palto S.P., Barnik M.I., Geivandov A.R., Kasyanova I.V., Palto V.S. (2015). Spectral and polarization structure of field-induced photonic bands in cholesteric liquid crystals. Phys. Rev. E.

[14] McConney M.E., Tondiglia V.P., Natarajan L.V., Lee K.M., White T.J., Bunning T.J. (2013). Electrically induced color changes in polymer-stabilized cholesteric liquid crystals. Adv. Opt. Mater..

[15] Xiang J., Li Y., Li Q., Paterson D.A., Storey J.M.D., Imrie C.T., Lavrentovich O.D. (2015). Electrically tunable selective reflection of light from ultraviolet to visible and infrared by heliconical cholesterics. Adv. Mater..

[16] Petriashvili G., Japaridze K., Devadze L., Zurabishvili C., Sepashvili N., Ponjavidze N., De Santo M.P., Matranga M.A., Hamdi R., Ciuchi F. (2013). Paper like cholesteric interferential mirror. Opt. Express.

[17] Shibaev P.V., Schlesier C. (2012). Distant mechanical sensors based on cholesteric liquid crystals. Appl. Phys. Lett..

[18] Mitov M. (2012). Cholesteric liquid crystals with a broad light reflection band. Adv. Mater..

[19] Hennig G., Brittenham G.M., Sroka R., Kniebühler G., Vogeser M., Stepp H. (2013). Bandwidth-variable tunable optical filter unit for illμmination and spectral imaging systems using thin-film optical band-pass filters. Rev. Sci. Instrum..

[20] Hikmet R.A.M., Kemperman H. (1998). Electrically switchable mirrors and optical components made from liquid-crystal gels. Nature.

[21] Li Y., Luo D., Peng Z.H. (2017). Full-color reflective display based on narrow bandwidth templated cholesteric liquid crystal film. Opt. Mater. Express.

[22] Jeong M.Y., Mang J.Y. (2018). Continuously tunable optical notch filter and band-pass filter systems that cover the visible to near-infrared spectral ranges. Appl. Opt..

[23] Lee K.M., Tondiglia V.P., Lee T., Smalyukh I.I., White T.J. (2015). Large range electrically-induced reflection notch tuning in polymer stabilized cholesteric liquid crystals. J. Mater. Chem. C.

[24] Takezoe H., Hashimoto K., Ouchi Y., Hara M., Fukuda A., Kuze E. (1983). Experimental study on higher order reflection by monodomain cholesteric liquid crystals. Mol. Cryst. Liq. Cryst..

[25] Ozaki R. (2019). Simple model for estimating band edge wavelengths of selective reflection from cholesteric liquid crystals for oblique incidence. Phys. Rev. E.

[26] Chou S.C., Cheung L., Meyer R.B. (1972). Effect of a magnetic fiedl on the optical transmission in cholesteric liquid crystals. Solid State Commun..

[27] Dumitrascu I., Dumitrascu L., Dorohoi D.O. (2006). The influence of the external electric field on the birefringence of nematic liquid crystal layers. J. Optoelectron. Adv. Mater..

[28] Jeon M.Y., Zhang J., Wang Q., Chen Z. (2008). High-speed and wide bandwidth Fourier domain mode-locked wavelength swept laser with multiple SOAs. Opt. Express.

[29] Ko M.O., Kim S.-J., Kim J.-H., Lee B.W., Jeon M.Y. (2014). Dynamic measurement for electric field sensor based on wavelength-swept laser. Opt. Express.

[30] Ko M.O., Kim S.-J., Kim J.-H., Jeon M.Y. (2018). In situ observation of dynamic pitch jumps of in-planar cholesteric liquid crystal layers based on wavelength-swept laser. Opt. Express.

[31] Jeong M.-Y., Wu J.W. (2010). Continuous spatial tuning of laser emissions with tuning resolution less than 1 nm in a wedge cell of dye-doped cholesteric liquid crystals. Opt. Express.

[32] Inoue Y., Hattori M., Kubo H., Moritake H. (2017). Faster pitch control of cholesteric liquid crystals. Jpn. J. Appl. Phys..

